# Divergent Human Cortical Regions for Processing Distinct Acoustic-Semantic Categories of Natural Sounds: Animal Action Sounds vs. Vocalizations

**DOI:** 10.3389/fnins.2016.00579

**Published:** 2017-01-06

**Authors:** Paula J. Webster, Laura M. Skipper-Kallal, Chris A. Frum, Hayley N. Still, B. Douglas Ward, James W. Lewis

**Affiliations:** ^1^Blanchette Rockefellar Neurosciences Institute, Department of Neurobiology & Anatomy, West Virginia UniversityMorgantown, WV, USA; ^2^Department of Neurology, Georgetown University Medical CampusWashington, DC, USA; ^3^Department of Physiology and Pharmacology, West Virginia UniversityMorgantown, WV, USA; ^4^Department of Biophysics, Medical College of WisconsinMilwaukee, WI, USA

**Keywords:** auditory system pathways, fMRI, mirror neuron system, acoustic communication, grounded cognition

## Abstract

A major gap in our understanding of natural sound processing is knowledge of where or how in a cortical hierarchy differential processing leads to categorical perception at a semantic level. Here, using functional magnetic resonance imaging (fMRI) we sought to determine if and where cortical pathways in humans might diverge for processing action sounds vs. vocalizations as distinct acoustic-semantic categories of real-world sound when matched for duration and intensity. This was tested by using relatively less semantically complex natural sounds produced by non-conspecific animals rather than humans. Our results revealed a striking double-dissociation of activated networks bilaterally. This included a previously well described pathway preferential for processing vocalization signals directed laterally from functionally defined primary auditory cortices to the anterior superior temporal gyri, and a less well-described pathway preferential for processing animal action sounds directed medially to the posterior insulae. We additionally found that some of these regions and associated cortical networks showed parametric sensitivity to high-order quantifiable acoustic signal attributes and/or to perceptual features of the natural stimuli, such as the degree of perceived recognition or intentional understanding. Overall, these results supported a neurobiological theoretical framework for how the mammalian brain may be fundamentally organized to process acoustically and acoustic-semantically distinct categories of ethologically valid, real-world sounds.

## Introduction

Critical to survival and social organization is our ability to view, hear, and understand the goals and intentions of others, including both human (conspecifics) and non-human (non-conspecific) animals. However, it remains unclear how the human auditory system is fundamentally organized to process the diverse range of natural, biologically relevant sounds, such as speech and action events, and provide the listener with a sense of meaning. In dual-stream models of sound processing dorsally directed cortical pathways, relative to primary auditory cortex (PAC), are involved in part with processing spatial information, such as localizing sounds and preparing motor movements or action schemas to engage or avoid the sound-source (Romanski et al., 1999; Rauschecker and Tian, 2000; Rauschecker and Scott, 2015). Conversely, ventrally directed cortical pathways, relative to PAC, are involved more in identification of sound patterns for perception of what the source is or its potential communicative content. This dorsal vs. ventral dichotomy has also been applied to models of spoken language processing (MacNeilage, 1998; Rauschecker and Scott, 2009; Arbib, 2010; Perlovsky, 2011; DeWitt and Rauschecker, 2013). In these models, dorsal pathways are generally implicated in temporal processing, such as amplitude or temporal envelope segmentation. In contrast, ventral pathways are involved in pattern matching for content, such as syllables, phonemes, and words. However, the processing that takes place along intermediate stages, between PACs and the higher-level semantic and audio-motor representations that are more commonly affiliated with recognition and behavior remains unclear. Thus, while hypothesized dorsal-ventral organizations for sound processing are reasonably well established, many gaps remain in our understanding of how the putative ventral pathways for sound recognition (“what is it”) may be organized to process the acoustic signal attributes that may be characteristic of different semantic or “acoustic-semantic” categories of natural sounds.

With regard to “bottom-up” theories of sound processing and perception, numerous animal models, and more recently human models, have revealed principles for how real-world sounds become processed subsequent to tonotopically organized regions (Rauschecker et al., 1995; Kaas and Hackett, 1998; Petkov et al., 2008; Lewis et al., 2009; Giordano et al., 2013). In particular, hierarchical models indicate that parallel processing pathways along auditory cortices have successive stages containing neurons that show increasing selectivity for complex spectro-temporal attributes (Fecteau et al., 2004; Medvedev and Kanwal, 2004; Kumar et al., 2007; Rauschecker and Scott, 2009; Schönwiesner and Zatorre, 2009; Lewis et al., 2012). Such organizations are suggestive of “matched-filter” tuning mechanisms, which may facilitate or mediate the detection of certain types of sounds or sound attributes that effectively segment sounds of interest from background noise (Suga, 1965; Pollak and Bodenhamer, 1981; Rose and Capranica, 1983; Lewicki, 2002; Woolley et al., 2005). However, there is no consensus as to what low- or high-level acoustic signal attributes comprise the basic building blocks for the processing and reconstruction of real-world, natural sounds as meaningful events to a listener.

Conversely, top-down driven models, such as embodied or grounded cognition models, have led to the development of theories of how various widespread brain regions may mediate semantic knowledge representations at a categorical level (Moore and Price, 1999; Barsalou et al., 2003; Miller et al., 2003; Hickok and Poeppel, 2004; Martin, 2007; Binder et al., 2009; Engel et al., 2009; Lewis et al., 2011; Bornkessel-Schlesewsky et al., 2015). However, many earlier human neuroimaging studies using human-produced sound stimuli, which in adult listeners tend to inherently entail complex semantic associations, likely recruited brain regions well outside of auditory cortex *proper* for processing higher order cognitive information, including linguistic features, subtle communicative nuances, and audio-motor schemas (Shallice, 1988; Grafton et al., 1997; Binder et al., 2000; Fiebach et al., 2003; Alain, 2007; Canessa et al., 2007; McNamara et al., 2008; Turkeltaub and Coslett, 2010; Meyer et al., 2011; Woods et al., 2011; Beer et al., 2013). Consequently, the cortical processing of highly familiar human-produced sounds may mask some of the more fundamental, or intermediate, stages of real-world, natural sound processing.

**Figure 1:**
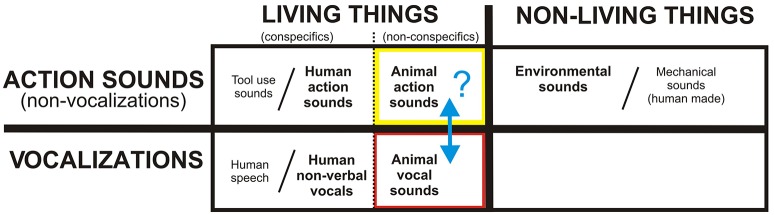
**A theoretical framework for the neurobiological organization of the human (and mammalian) brain for processing and recognizing different acoustic-semantic categories encompassing all real-world, natural sounds**. Human speech, tool use sounds, and human-made machinery sounds are represented as extensions of categories in this rudimentary model, while music and emotionally-laden sounds are regarded as higher forms of communication. The present study is testing the putative boundary (blue double headed arrow) between action sounds (yellow box) vs. vocalizations (red) using animal (non-conspecific) sound stimuli. Refer to text for other details.

In the present study, we sought to further bridge bottom-up and top-down models of fundamental aspects of sound recognition by examining functional networks for processing two categories of semantically (and acoustically) distinct real-world sounds produced by non-human animals: Action sounds vs. vocalizations. Such sounds would arguably be ethologically valid, yet have fewer complex semantic associations than human-produced sounds in general. More specifically, based on a large number of earlier neuropsychological and neuroimaging studies of hearing perception, we sought to test a general theoretical framework for how the mammalian brain may be, or may become, optimized for processing real-world sounds at a semantic or acoustic-semantic categorical level (Figure 1). This simple model incorporates the classical living vs. non-living semantic category boundary for word-form knowledge as one major division (Warrington and Shallice, 1984; Hillis and Caramazza, 1991; Capitani et al., 2003) but here being applied more specifically to sound-sources. A second major theorized division, being explicitly tested in the present study, distinguishes vocalizations from action sounds (non-vocalizations). Earlier studies of speech and human vocalization processing have identified the superior temporal gyri (STG) and surrounding territories as principle “intermediate” cortical processing stages for vocal communication sounds (Belin et al., 2000; Turkeltaub and Coslett, 2010), including a left lateralized extended network for intelligible speech sounds (Ohnaka et al., 1995; Scott et al., 2000, 2006; Engelien et al., 2006; Friederici et al., 2010) and right lateralized extended network preferential for processing non-verbal and prosodic communication sounds (Corballis, 1989; Zatorre et al., 1992; Thierry et al., 2003; Gandour et al., 2004). The semantic boundary for processing vocalizations produced by animals vs. humans (i.e., non-conspecifics vs. conspecifics) has received relatively less attention (Figure 1, black dotted line next to red box). Nonetheless, at least a subset of the above mentioned STG foci and surrounding cortical regions are reported to be recruited when human listeners process animal (non-conspecific) vocalizations (Maeder et al., 2001; Lewis et al., 2005, 2009; Engelien et al., 2006; Altmann et al., 2007; Belin et al., 2008; Doehrmann et al., 2008; Talkington et al., 2012, 2013). This conspecific vocalization processing boundary concept is generally consistent with neuroimaging studies of other mammalian species, including monkeys (Kohler et al., 2002; Petkov et al., 2008) and canines (Andics et al., 2014), which reveal regions or pathways preferential for processing their respective conspecific vocalizations and calls. Consequently, for the present study we regarded human vocalizations as belonging to a distinct acoustic-semantic category, which potentially facilitates recruitment of down-stream cortical pathways for additional processing of communicative intent or other complex learned semantic knowledge associations. Therefore, human produced vocalization sounds were not included in order to target more rudimentary or intermediate stages of sound processing.

With regard to action (non-vocalization) sounds, we previously reported the existence of a four-fold dissociation of cortical networks for processing different categories of real-world sounds, distinguishing those produced by human, animal, mechanical, and environmental sources (Engel et al., 2009; Lewis et al., 2011). These studies revealed the left and right posterior insulae as regions preferential for processing non-human animal action sounds. Thus, also included in our theoretical framework (Figure 1, black dotted line next to yellow box) is a semantic processing boundary between human vs. animal action sounds. On this front, our model incorporates grounded (“embodied”) cognition theories, which at least loosely relate to mirror neuron systems (MNS), which may reflect system-level processing strategies for matching heard action sounds to the listeners own repertoire of sound-producing motor actions; this processing strategy is thought to provide a listener with a sense of meaning or intention behind the sound-producing action (MacNeilage, 1998; Arbib, 2001; Buccino et al., 2001; Barsalou et al., 2003; Rizzolatti and Craighero, 2004; Bidet-Caulet et al., 2005; Calvo-Merino et al., 2005; Gazzola et al., 2006; Lewis et al., 2006; Lahav et al., 2007; Martin, 2007; Mutschler et al., 2007; Barsalou, 2008; Galati et al., 2008; Giordano et al., 2010; Imai et al., 2015). Most studies examining biological motion (visual or auditory) have focused on using human (conspecific) stimuli (for review, see, Chouchourelou et al., 2012). However, one study using point-light animations of both human and animal biological motion, together with brain lesion mapping, reported cases of selective impairment in recognizing human motion but not animal motion (Han et al., 2013), thereby distinguishing these semantic action-event categories. Ostensibly, when processing biological sounds that cannot be fully or accurately mimicked motorically by one's own body or limbs (e.g., a horse galloping or pigeon flapping its wings) the brain must still attempt to process and decode the acoustic signals for potential meaning behind the heard action. Thus, in the present study we also regarded human vs. animal action sounds as belonging to semantically and neurobiologically distinct categories.

At a gross level, our model of acoustic-semantic category-level sound processing makes a strong prediction for the existence of a major divergence in cortical pathways for processing animal action sounds vs. animal vocalizations (Figure 1, blue arrow). As alluded to earlier, higher level cognitive systems tend to show greater hemispheric lateralization for information processing (Preuss, 2011), with some dependence on task and developmental plasticity (Hamzei et al., 2003; Iacoboni et al., 2005; Lewis et al., 2006). This lateralization effect is especially prominent with processing associated with language, vocal and instrumental music, and other complex semantic-level processing that entails potentially higher forms of acoustic communication or thought (Peretz and Zatorre, 2005; Binder et al., 2009; Meyer et al., 2011; Alluri et al., 2013; Cai et al., 2013). Hence, our presumed rudimental model for the acoustic-semantic organization of sound processing further predicts that the processing of non-conspecific animal sounds should recruit divergent pathway activation in *both* cortical hemispheres that likely relate to relatively less specialized processing weighted by bottom-up acoustic signal processing. Thus, the goal of the present study, using fMRI, was to test for the existence of divergent cortical processing streams along human auditory cortices, presumably in both hemispheres, that respect the theorized acoustic-semantic boundary for processing *non-conspecific animal* action sounds vs. vocalizations as ideal stimulus sets to critically test this putative major boundary—which may represent a sensory processing boundary common to most if not all mammals. Exploring and establishing fundamental cortical organizational principles behind natural sound processing is ultimately expected to help advance both anthropological theories of spoken communication evolution, as well as neurodevelopmental models of hearing perception and spoken language acquisition in healthy young children and those with speech or language delays.

## Materials and methods

### Participants

We tested 17 right-handed participants (Avg. 23.6 ± 2.6 years, range 20–29, 8 female) who performed one or multiple paradigms using functional magnetic resonance imaging (fMRI). All participants were native English speakers with no history of neurological or psychiatric disorders, and a self-reported normal range of hearing with no auditory impairments. Informed consent was obtained for all participants following guidelines approved by the West Virginia University Institutional Review Board and in compliance with the Code of Ethics of the World Medical Association.

### Sound stimulus creation and presentation

The sound stimulus set consisted of 29 animal vocalizations and 29 animal action sounds (Table 1; also see art piece in Supplemental Figure S4) derived from professional compilations (Sound Ideas, Inc., Richmond Hill, Ontario, Canada), incorporating recordings of animals in isolation with little to no background noise. The animal vocalizations selected were produced by vibrations of the vocal folds, primarily of various land mammals. To facilitate data interpretation, we minimized the use of bird calls and aquatic mammals, due to differences in their mechanisms of sound production via their respective “vocal” systems (e.g., syrinx or blowhole), as a listener's ability to mimic sounds may influence perceptual processing mechanisms (Liberman and Mattingly, 1985). Animal vocalizations were initially derived from another study by our lab examining mimicry (Talkington et al., 2012) in which at least 46 of 50 participants could correctly categorize the sound as non-human (in contrast to animal vocalizations mimicked by a human; two alternative-forced choice [2AFC] task). Animal action sounds were also selected based upon whether they were produced primarily by land animals and were deemed to be devoid of any vocalization content. This set of sounds was initially derived from our earlier study (Engel et al., 2009), which included action sound sources that were categorized as animal (vs. human, mechanical, or environmental; four alternative forced choice [4AFC]) by more than 80% of participants.

**Table 1:** **List of sound stimuli together with some of their acoustic signal attributes and perceptual features**.

**Animal Action sounds**	**HNR**	**WE**	**SSV**	**Intent**	**Recog**	**Emotion**
Bat flapping wings	2.83	−5.40	1.52	2.00	2.15	−0.07
*Bird, flying away*						
Cow(s) moving in pen	2.23	−3.39	1.43	2.10	2.65	0.07
Dog (large) eating food from bowl	−1.71	−3.21	1.81	2.90	2.20	0.53
*Dog (small) eating food*						
Dog drinking water from bowl	1.27	−4.28	2.12	3.20	2.60	0.67
Dog footsteps	−0.68	−1.94	0.38	2.65	2.60	0.07
Dog licking/lapping	−1.52	−2.49	1.53	2.80	2.20	0.33
*Dog panting heavily*						
Dog shaking head/ears flapping	−0.93	−3.50	0.79	2.80	2.80	0.07
Dog sniffle and digging	−1.01	−2.96	0.80	3.15	3.15	0.53
Dog swimming and shakes	−4.00	−2.37	0.32	2.90	2.95	0.07
Dog trotting, toe nails on floor #1	−0.64	−2.53	0.41	2.75	2.70	0.33
Dog trotting, toe nails on floor #2	−1.24	−3.59	0.73	2.90	3.10	0.80
Dog/animal licking & chewing	−1.40	−2.54	1.52	2.75	2.35	0.27
Horse eating	−0.74	−4.58	1.84	2.30	1.85	0.40
Horse galloping #1	−1.65	−6.17	1.91	2.95	3.20	0.07
Horse galloping #2	3.16	−2.97	2.03	2.90	3.40	−0.07
Horse galloping #3	1.52	−2.60	1.72	3.15	3.45	0.13
Horse galloping #4	3.88	−3.28	2.28	3.15	3.45	0.33
Horse trotting	2.80	−3.38	2.19	3.30	3.65	0.33
Horse walking #1	7.72	−6.02	2.95	3.50	3.75	0.40
Horse walking #2	2.33	−2.87	0.80	3.35	3.40	0.33
Horse walking on cobblestone	1.14	−4.88	1.77	3.25	3.85	0.60
Pigeon flight	−3.67	−2.30	0.36	2.65	2.75	−0.13
*Rattlesnake rattling #1*						
*Rattlesnake rattling #2*						
*Rattlesnake rattling #3*						
Zebra trotting on dirt	3.59	−3.21	2.54	3.05	3.50	0.60
Average	0.58	−3.50	1.47	2.89	2.94	0.29
StDev	2.74	1.18	0.76	0.38	0.57	0.26
Baby bear bark	3.84	−6.90	4.29	2.45	2.30	−1.00
Bear call #1	1.64	−8.89	2.88	2.55	2.50	−0.73
Bear call #2	4.50	−7.96	2.95	2.90	3.25	−0.93
Bear call #3	12.52	−8.25	5.15	2.50	2.75	−0.93
Bear growl #1	−1.43	−8.64	1.51	2.70	2.50	−1.40
Bear growl #2	1.38	−8.40	1.28	2.55	2.20	−1.07
Bear growl #3	1.59	−6.57	0.86	3.05	2.95	−1.60
Bobcat growl	−1.14	−6.47	1.34	3.00	2.70	−1.40
Bull call #1	12.08	−11.25	0.92	2.45	2.75	−0.93
Bull call #2	4.74	−8.19	1.87	2.75	2.65	−1.73
Bull call #3	11.18	−9.86	3.25	2.40	3.50	−0.47
Camel lowing	13.05	−10.00	0.82	1.95	2.60	−1.13
Camel moo	13.01	−10.00	0.78	2.35	2.45	−0.87
Cougar growl	1.83	−7.41	3.71	3.60	3.55	−1.73
Cow moo	16.90	−10.24	1.90	2.05	2.90	−0.80
Dog bark	4.03	−7.51	3.21	3.45	4.00	−0.20
Dog growl-sigh	4.43	−7.01	3.87	2.90	3.10	−0.87
Dog whine and bark	6.27	−7.44	2.41	3.40	3.60	−1.33
Goose (Canadian) honks	9.64	−7.99	9.63	2.45	3.40	0.53
Horse whinny	8.64	−6.14	1.03	3.45	3.90	−1.07
Jackal call	17.79	−7.14	4.29	2.90	3.30	−0.47
Lion roar	1.69	−10.82	2.30	3.00	3.45	−1.00
Monkey scream #1	14.96	−6.20	4.39	3.05	3.75	−0.93
Monkey scream #2	10.08	−5.92	0.88	3.00	3.70	−1.67
Monkey vocal	14.07	−7.37	6.76	2.65	3.75	1.00
Pig squeal	6.70	−5.25	0.69	3.10	3.70	−1.73
Sheep baa	8.46	−5.70	16.66	2.65	3.95	0.60
Wolf bark	9.42	−8.54	2.85	3.35	3.70	−0.60
Wolf growl	1.13	−5.80	0.61	3.35	2.80	−1.47
Average	7.34	−7.86	3.21	2.83	3.16	−0.89
StDev	5.51	1.62	3.30	0.43	0.55	0.69

*Italicized entries were censored a priori from fMRI data analyses. HNR = harmonics-to-noise ratio; WE = Weiner entropy; SSV = spectral structure variation; Recog = recognition. Refer to text for other details*.

Using commercially available software (Adobe Audition 3.0, Adobe Systems Inc.), the two categories of sound stimuli were carefully matched for duration (2.7 ± 0.2 s) and total root mean squared power (−17.6 ± 0.5 dB), which remained matched both before and after *post-hoc* censoring six of the action sounds from data analyses (addressed below). In addition, the onsets/offsets were ramped by 25 ms to avoid “pops” during sound presentation. Sound stimuli were converted to one channel (mono, 44.1 kHz, 16-bit) but presented to both ears via ear buds, thereby removing any binaural spatial cues.

To validate the specificity and clarity of the sound source category, five participants who were not included in the fMRI studies, and were naïve to the purpose of the experiment, assessed the censored sound stimuli to (1) verify that the animal vocalizations and animal action sounds were easily categorized as an action or vocalization, and (2) verify that sounds were clearly perceived as not being produced by a human agent. Sounds that were unclear or retained ambiguous elements that might cross the theorized category boundary/boundaries were replaced by other examples until a complete set of 58 sound stimuli was obtained that all five listeners could unanimously categorize correctly. After obtaining post-scanning feedback from several fMRI participants, we determined that some of the action sounds were perceived as possibly containing overt communicative intent (e.g., rattlesnake rattling to indicate “stay away”), human intervention (e.g., hearing metallic sounds of a dog collar jingling), or were “breathy” (e.g., panting) some participants confused as blending across the conceptual boundary between action events and vocalizations. Consequently, we censored out fMRI responses (blood oxygen-level dependent; BOLD) activation brain responses to six of the animal action sounds *post-hoc* (Table 1, italicized entries). Note, that including or excluding these sound stimuli did not qualitatively affect the main results (data not shown), thought these exclusions facilitated data interpretation.

### Acoustic signal features of the sound stimuli

The sound stimuli were assessed for differences in quantifiable high-order acoustic signal attributes and fine scale power spectra using freely available phonetic software (Praat, http://www.fon.hum.uva.nl/praat/, version 5.1.04). The animal action sounds generally had greater power at the lower frequency ranges (long term average spectrum at 10 Hz bandwidth increments). This included a significantly greater prevalence of amplitude fluctuations at rates between 10 and 50 Hz for the animal action sounds [single factor ANOVA *F*_(1, 9)_ = 473, *p* < 10^−8^; see Supplemental Figure S1A], which is a range typically regarded as envelope information (2–50 Hz; also known as “amplitude envelope” or “temporal information”) in speech literature (Rosen, 1992). Some of this difference in power at lower frequencies likely reflected temporal envelope events, such as the pace of locomotion sounds (e.g., footsteps and galloping) and temporal harmonics therein. Several low- and high-order acoustic signal attributes could distinguish the two semantic categories of sound on average (see Table 1). For instance, the global harmonics-to-noise ratio (HNR) value was determined for each sound using Praat software (10 ms time step; minimum pitch cutoff of 75 Hz, 20 kHz ceiling; and 1 period per window) and on average revealed a significantly greater degree of harmonic structure for the animal vocalizations (animal vocalizations = +7.34 dB_HNR_, animal action sounds = +0.58 dB_HNR_; two-tailed *t-test, p* < 10^−7^), which was expected based on earlier studies (Lewis et al., 2005, 2009). Additionally, as a measure of spectral flatness, which has also been used to study the dynamics of vocal imitation in song birds (Tchernichovski et al., 2001), we assessed the magnitudes of signal entropy (Wiener entropy, WE). Entropy measures, and related spectral structure variation (SSV) measures addressed below, were derived using a freeware script with Praat software (“Wiener entropy,” see online supplemental software script). On average, action sounds showed a significantly greater magnitude in signal entropy: Vocalization = −3.50, action sounds = −7.86; two-tailed *t-test, p* < 10^−15^). Using a measure of change in entropy over time (an index of spectral dynamics or rate of change of spectral structure), the animal vocalizations showed on average a greater degree of SSV measures (vocalizations = 3.21, action sounds = 1.47; two-tailed *t-test, p* < 0.01). Each of the above signal attributes were assessed and examined here because they have been implicated in auditory stream segregation and auditory object perception in earlier studies (Lewis et al., 2009, 2012; Reddy et al., 2009). We also assessed potential category-level differences in modulation power spectra (MPS), using techniques that have revealed spectro-temporal attributes of sound that may be processed along distinct pathways in auditory cortices in humans and macaques (Woolley et al., 2005; Cohen et al., 2007; Elliott and Theunissen, 2009; Kuśmierek et al., 2012; Herdener et al., 2013; Santoro et al., 2014). We used freely available Matlab software programs to derive MPS measures (http://strfpak.berkeley.edu), using a 32 Hz frequency band and low-pass filter method for the ensemble of 23 animal action sounds and 23 (of the 29) animal vocalizations (Supplemental Figure S1C). In short, various quantifiable low- and high-order acoustic signal attributes could distinguish the two semantic categories of sound on average (action sounds vs. vocalizations). Consequently, we referred to the real-world natural categories of sound in Figure 1 as “acoustic-semantic” categories. We further conducted *post-hoc* analyses of differentially activated brain regions to test for activation that showed parametric sensitivity to several of the above acoustic signal attributes (addressed below), as well as perceptual features, thereby probing for their potential functional roles.

### Perceptual features of the sound stimuli

To identify potential perceptual features that may influence cortical processing of the sound stimuli, we assessed the perceived intention behind the sounds, the degree to which they could be recognized, and the emotional valence associated with each sound. Using freely available PsychoPy2 (v1.73.05) software (Peirce, 2007), a separate set of non-imaging listeners (*n* = 20, 10 female) heard all sounds in a random order while in a sound isolation booth, and rated them on a 1-4 Likert scale with the instructions: “With regard to the sound you heard, your ability to recognize the Intention was: (1) almost certainly not, (2) unlikely, (3) likely, to (4) almost certainly”. While there was a full range of ratings, overall there were no significant differences in mean ratings of intent between the two categories of sound (Table 1, *t-test*, two-tail *p* < 0.58). Using a counter-balanced design, these participants also separately rated each sound for their ability to recognize the sound with the instructions: “With regard to the sound you heard, your ability to Recognize it was: (1) almost certainly not, (2) unlikely, (3) likely, to (4) almost certainly.” Again, there was a full range of responses but no significant differences in mean ratings between categories (*t-test*, two-tail *p* < 0.16).

To assess the emotional valence of the sounds, another group of listeners (*n* = 15, 8 female) rated all the sounds, presented in a random order (using PsychoPy software), on a 5-point scale of perceived emotional valence. This ranged from very negative (−2), to neutral (0), to very positive (+2) for the degree of emotional content ascribed to the agent (animal) producing the action sound or vocalization. The animal vocalizations were rated (Supplemental Figure S1B) as having a relatively negative emotional valence overall (Mean/*SD* = −0.89 ± 0.69) while animal action sounds were rated closer to neutral valence (+0.29 ± 0.26), which were ratings that on average significantly differed from one another [single factor ANOVA, *F*_(1, 51)_ = 61.3, *p* < 10^−13^].

### Scanning paradigms

#### Sound categorization paradigm

The main auditory scanning paradigm for fMRI imaging (*n* = 17, 8 female) consisted of two separate runs that were to be presented three times each. Across the two runs, the 58 sound stimuli plus 24 silent events were presented in pseudo-random order, with no more than two silent events presented in a row. This randomized approach was adopted to avoid or minimize activation related to state-dependent effects observed in block designs (Rehme et al., 2013). The high-fidelity sound stimuli were delivered using a Windows PC computer, with Presentation software (version 11.1, Neurobehavioral Systems Inc.) via a sound mixer and MR compatible ear buds (Model S14, Sensimetrics Corp., Malden MA). Stimulus loudness was set to a comfortable level for each participant: Immediately after scanning a computer generated 1 kHz pure tone was played through the sound transmission system and the intensity was typically in the range of 80–83 dB C-weighted to each ear (Brüel & Kjær 2239a sound meter). Participants were instructed to determine by 2AFC left hand button response whether the sound heard was (1) an animal vocalization (index finger) or (2) an animal action sound (middle finger). Although none of the participants had any previous exposure to these specific sound stimuli, they were presented with several of the sound stimuli during a brief practice fMRI scan to ensure the instruction were clearly understood, and they were explicitly informed that all the sounds they heard would be animal actions and animal vocalizations.

#### Button press control paradigms

To identify brain regions that may be involved in the preparation and mechanics of button presses, we conducted two motor output-related control paradigms germane to the main auditory paradigm. As a first control condition, one participant performed the main auditory paradigm using her right hand for button presses for two runs and her left hand for two runs. This allowed for a direct comparison of the anticipated effects of primary motor cortex activation related to the hand used when responding. As a second control condition, two of the participants performed a separate button press paradigm after the main sound categorization task. This involved a simple ON/OFF block design cued by short tone pips every 20 s for 4 ON and 5 OFF blocks. During the ON cycles, they made self-initiated button box responses with their left index or middle finger at approximately the same rate as during the main auditory scanning paradigm. ON cycles were flanked by OFF cycles during which they would rest their hand.

#### Magnetic resonance imaging and data analysis

The fMRI imaging paradigms were all conducted on a 3 Tesla Siemens Verio MRI scanner using a 32-channel head coil. For all paradigms, participants kept their eyes closed. For the auditory paradigm, a sound or silent event was presented every 10 s, and BOLD signals were collected continuously using an echo planar pulse sequence (ep2d: TR = 2000 ms, TE = 30 ms, FOV = 240 mm, 75° flip angle). Whole-head brain volumes were collected, including 32 axial slices at 4 mm slice thickness (plus 0.6 mm gap) and 3.75 × 3.75 mm^2^ in-plane resolution. Continuous scanning, as opposed to sparse sampling, was utilized in order to obtain BOLD signals over time that could be more robustly analyzed using gamma-variate models. Sound stimuli were heard over continuous scanner noise. The presentation of each auditory stimulus event was triggered by the fifth transistor-transistor logic (TTL) pulse from the MRI scanner for each TR (triggering sound or silent events every 10 s), thereby ensuring accurate time stamps for image acquisition relative to stimulus sound onsets and for recording button press reaction times. After the completion of the functional imaging scans, whole brain T1-weighted anatomical MR images were collected using an MPRAGE pulse sequence (1.5 mm sagittal slices, 0.625 × 0.625 mm^2^ in-plane resolution, TI = 1100 ms).

All functional datasets were processed using Analysis of Functional NeuroImages (AFNI) and associated software plug-in packages (http://afni.nimh.nih.gov/) (Cox, 1996). For each paradigm, the 20th volume of the final functional scan, closest to the anatomical image acquisition, was used as a common registration image to globally correct motion artifacts due to head translations and rotations using program 3dvolreg. Voxels were subjected to a Gaussian spatial blurring of 8 mm (Mikl et al., 2008), and BOLD signals were then converted to percent signal change on a voxel-wise basis relative to BOLD signal during silent events for each scanning run for each participant.

For subsequent group-level analyses we conducted both volumetric and surface-based alignment methods. Surface-based alignment methods generally enable greater accuracy in the localization of activated ROIs across the cortical folds, such as along the superior temporal plane and fronto-parietal operculum (Desai et al., 2005). However, both alignment methods yielded qualitatively similar results for our datasets, so only the results from the standard Talairach volumetric alignment method are presented here. Individual anatomical brain volumes were manually aligned to standardized Talairach space (Talairach and Tournoux, 1988). For each individual, scanning runs were concatenated into a single time series and corrected for baseline linear drifts (six runs for fourteen participants, five runs for one participant, and four runs for two participants). A multiple linear regression analysis was performed using a gamma-variate waveform model (using AFNI program 3dDeconvolve) of the sound onsets for each category of sound stimulus to compare cross-categorical BOLD brain responses (animal vocalizations vs. animal action sounds), both relative to averaged BOLD signals from silent events as a baseline control (used for computing percent signal changes). We further restricted the analysis to reveal only those contrasts where the averaged BOLD activation responses to vocalizations or action sounds, or both, were positive relative to the responses to baseline silent events.

For the primary analyses, only those imaging events that corresponded to correctly categorized sounds for a given participant (as determined by button press accuracy) were retained for analyses (average 96.9% accuracy for vocalizations and 94.3% for action sounds across all scanning runs, rejecting trials with omissions or commissions. There were insufficient numbers of incorrectly categorized sounds for statistical error-trial analyses. The resulting BOLD multiple regression coefficients (statistical maps) were spatially low-pass filtered (6 mm box filter) and subjected to *t-test* and thresholding. We also processed all individual datasets using no spatial blurring, which yielded qualitatively similar results but required lower group-averaged threshold settings (data not shown).

**Figure 2:**
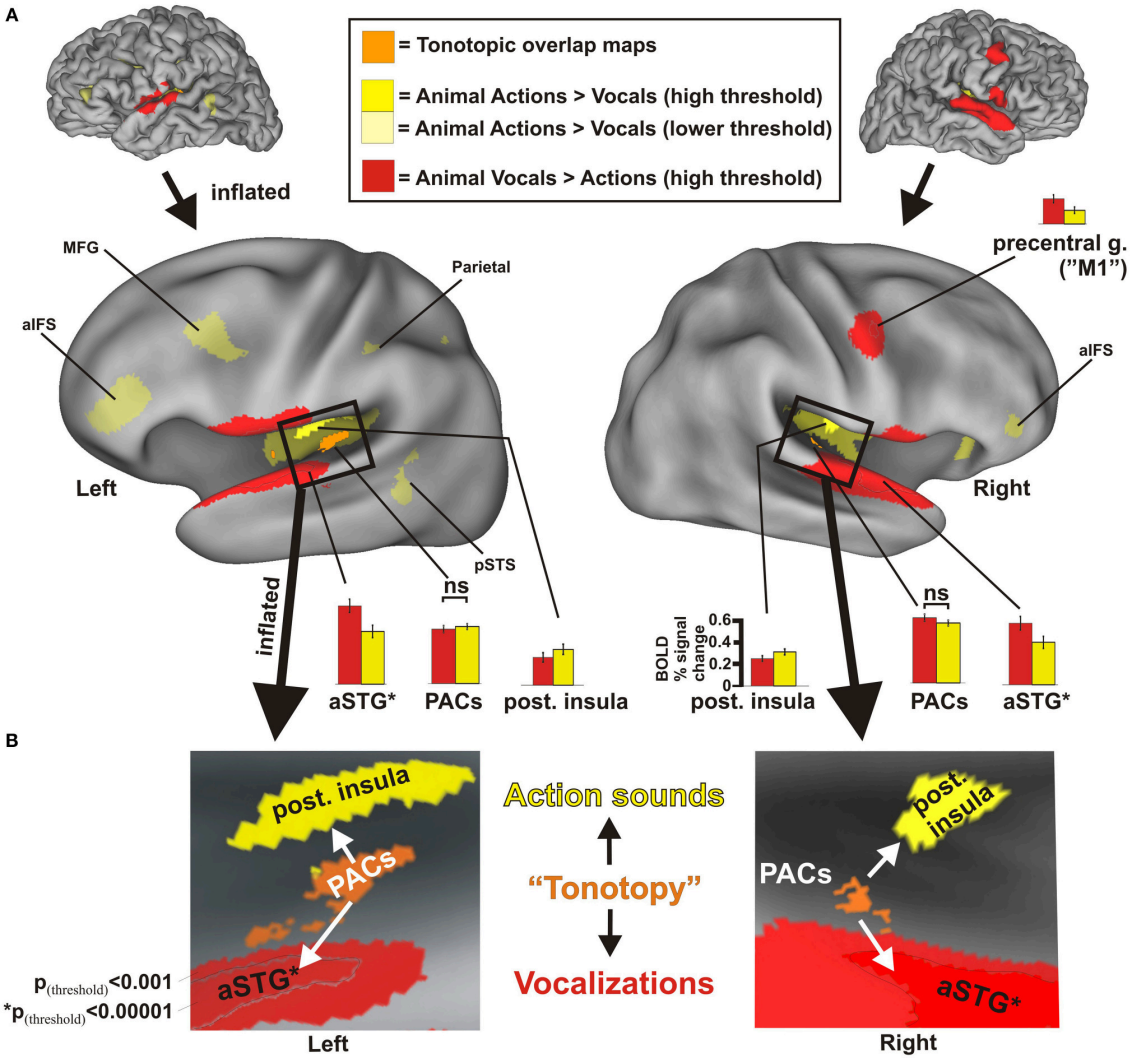
**Results from group-averaged fMRI data (***n*** = 17) revealing divergent cortical pathways for processing animal action sounds (yellow) vs. animal vocalizations (red), both relative to processing silent events (solid yellow hues at high threshold settings: ***p***_**(uncorr)**_ < 0.001, alpha corrected for multiple comparisons to ***p***_**(corr)**_ < 0.05; transparent yellow hues at a lower threshold setting ***p***_**(uncorr)**_ < 0.05, ***p***_**(corr)**_ < 0.05)**. **(A)** Data are illustrated on fiducial and inflated PALS atlas surface models. For comparison, functionally defined estimates of tonotopically organized primary auditory cortices (PACs, orange cortices) are also mapped (refer to Methods). Histograms indicate the BOLD percent signal changes (average ± SEM) in response to each sound category relative to silent events for various regions of interest, all of which were significant at *p*_(corr)_ < 0.001 except for the PAC regions. aIFS = anterior inferior frontal sulcus, aSTG = anterior superior temporal gyrus, M1 = primary motor cortex, MFG = medial frontal gyrus, PACs = primary auditory cortices, pSTS = posterior superior temporal sulcus. aSTG^*^ = foci (black outline) based on *p*_(corr)_ < 0.00001 were used for generating histogram data more highly restricted to the aSTG to facilitate data interpretation. ns = not significant. **(B)** Insets illustrate boxed regions from the 3D models in Panel **(A)** after further inflation and flattening, which highlight the main findings from this study (all data at *p*_(uncorr)_ < 0.001, *p*_(corr)_ < 0.05). Refer to text for other details.

**Figure 3:**
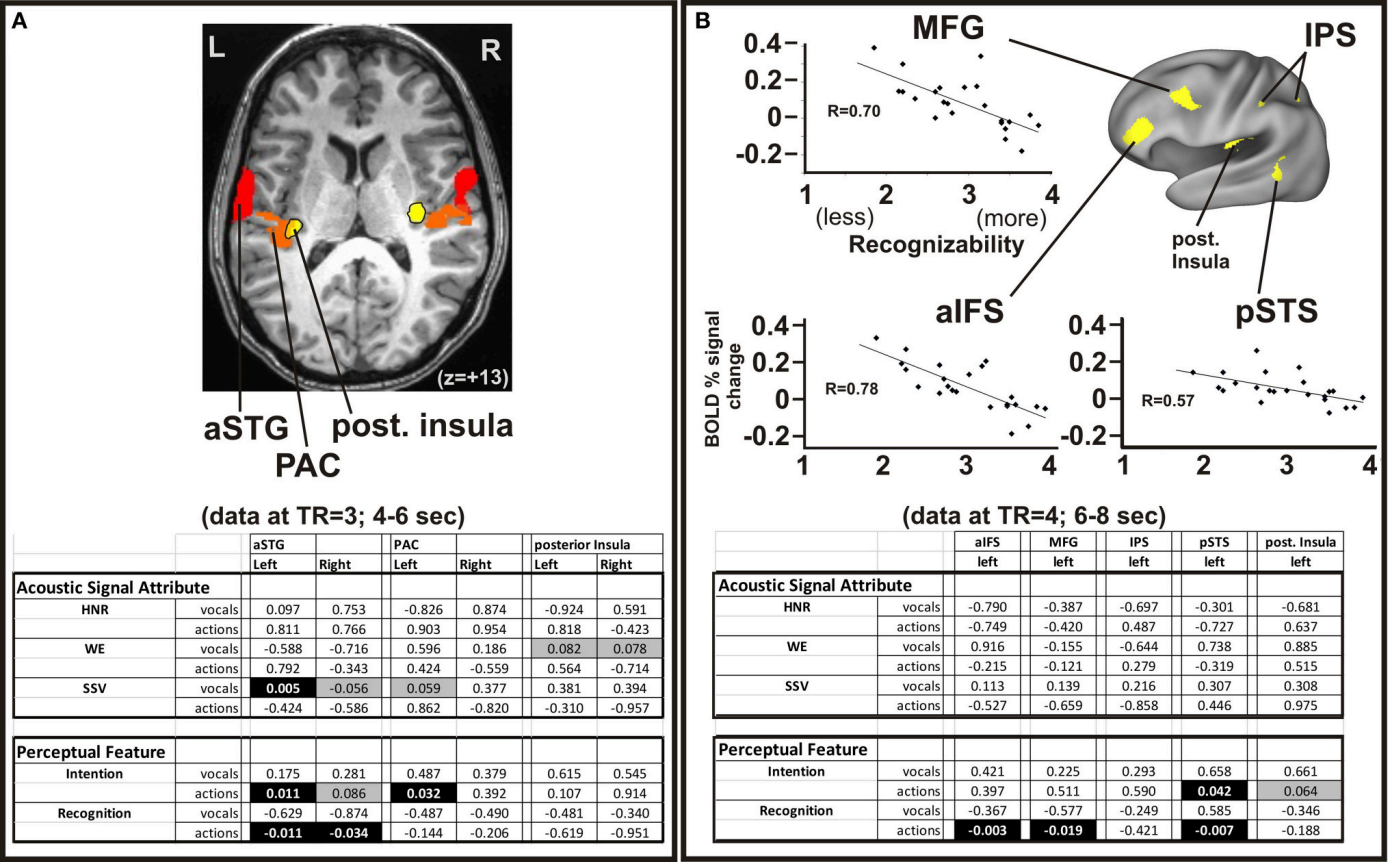
**Parametric activation analyses related to the acoustic signals and perceptual attributes of the animal sounds. (A)** The group-averaged datasets from Figure 2 are superimposed on an axial section of one participant (Talairach z = +13) showing the divergent processing regions (yellow vs. red) in both hemispheres. Multiple linear correlation *p*-value fits between regions of interest with the quantifiable high-order acoustic signal attributes (HNR, WE, SSV) and perceptual features (Intention, Recognition) of the animal action sounds and vocalizations (from Table 1) at the brain imaging time phase of TR = 3, occurring 4–6 s after sound onset. Negative correlations are indicated by negative value entries. Cells with black highlighting depict *p* < 0.05 (significant), and cells with gray highlighting depict *p* < 0.09 (trending). **(B)** Top panels show linear correlation charts for three of the five left hemisphere ROIs (MFG, aIFS, and pSTS; also evident in Figure 2). Charts illustrate BOLD percent signal change activation relative to acoustic signal attributes and perceptual ratings as in **Panel A**, except at the brain imaging time phase of TR = 4, occurring 6–8 s after sound onset. Lower panel shows results from a multiple linear regression analysis (correlation *p*-value fits) for all five ROIs sensitive to animal action sounds. Refer to Methods for further details.

To correct for multiple comparisons (multiple inference problem) (Forman et al., 1995; Eklund et al., 2016), we used AFNI related software. We estimated spatial blurring in our data by examining signal noise from the residual error time series from the full model of two individual's input data runs (3dDeconvolve software), blurring the error signals at 6 mm (roughly 1.5 times the size of acquisition voxels). This yielded estimated full-width half-max Gaussian filter widths of x = 6.5, y = 6.5, and z = 6.4 mm spatial smoothness. A brain mask was created for each of the subjects (3dAutomask software; dilation factor of 2). Using the brain mask and the above spatial filter widths, 10,000 Monte Carlo simulations were applied using 3dClustSim (version 16.2.06) to obtain corrected significance levels. For data in Figures 2, 3, a combination of a minimum cluster size threshold (6.4 voxels, or 414 mm^3^ volume; two-sided thresholding) and an individual voxel probability threshold (*p*_thr_ < 0.001) achieved a specified overall false detection probability (i.e., family-wise error rate; FWER) of alpha = 0.05, [*p*_(corr)_ < 0.05], which was herein designated as the high threshold setting. A lower threshold setting was also used to reveal subtler effects, which was obtained with a cluster size of 44.6 voxels (2885 mm^3^ volume) and *p*_thr_ < 0.05, which also yielded a corrected significance level of *p*_(corr)_ < 0.05. Functional imaging data were transformed into standardized Talairach coordinate space and for visualization purposes were projected onto the PALS atlas cortical surface models (in AFNI-Talairach space) using Caret software (http://brainmap.wustl.edu) (Van Essen et al., 2001; Van Essen, 2005).

#### Multiple linear regression analyses

Several *post-hoc* linear and multiple linear regressions analyses were used to identify correlations between various acoustic signal attributes (Table 1) or perceptual features of the sound stimuli (within and across categories) with activation strength of a voxel-wise basis for various regions of interest (ROIs). We modeled the BOLD signal as the linear convolution of the input stimulus (whether vocal sounds or action sounds), with the hemodynamic response function (HRF) for that particular stimulus. This BOLD signal was in addition to the baseline signal, which was modeled as a 3rd order polynomial, which was fit separately for each of the six concatenated runs. This resulted in the following multiple linear regression model for the fMRI signal:

$$\begin{array}{l} y(t)=\;\sum_{k=1}^{6}[b_{0,k}+b_{1,k}(t-t_{0,k})+b_{2,k}{\left(t-t_{0,k}\right)}^{2}\\ \;+b_{3,k}{\left(t-t_{0,k}\right)}^{3}]I_{k}(t)+\;\sum_{i=0}^{15}h_{VS}(i)\;VocalSounds\;(t-i)\\ \;+\sum_{j=0}^{15}h_{AS}(j)\;ActionSounds\;(t-j)+\varepsilon (t) \end{array}$$

where *y(t)* = measured fMRI signal (six concatenated runs), *k* = run index, *t*_0, k_ = time at beginning of *k*th run, *I*_k_(*t*) = indicator function for *k*th run (i.e., *I*_k_(*t*) = 1 if *t* occurs during *k*th run, 0, otherwise), *h*_VS_(*i*) = HRF for vocal sounds, *h*_AS_(*j*) = HRF for action sounds. Least squares fitting of the above model allowed for testing of various hypotheses concerning the fit coefficients, such as testing for significance using the above signal time series as well as testing at two distinct temporal phases of BOLD acquisition time, including the TR's corresponding to 4–6 s and 6–8 s after sound onset, respectively.

#### Reaction time data analyses

The average response times to correctly categorized vocalizations across all scanning runs was 2.26 ± 0.25 s and to correctly categorized (and *post-hoc* censored) animal action sounds was 2.26 ± 0.28 s, showing no significant difference [ANOVA *F*_(1, 157)_ = 0.014, *p* < 0.90]. To characterize potential within-session changes in task performance, we analyzed both reaction time and accuracy response data. On average the reaction times to categorizing the sounds during the first two of six runs vs. the last two runs showed changes across the scanning session: For animal vocalizations was 2.53 s and then 2.35 s respectively (*n* = 13 subjects; two tailed *t-test, p* < 0.0013), and for animal action sounds was 2.51 and then 2.35 s (*p* < 0.0011). The first two runs for vocalization sounds yielded 97.6 vs. 97.6% accuracy (no difference) in the last two runs, while animal action sounds yielded 98.0 vs. 96.0% (two-tailed *t-test, p* > 0.15, not significant) respectively. Thus, while there was some reaction time improvement over the course of the experiment and some mild perceptual deterioration effects over time (Mednick et al., 2005), participants were effectively performing at ceiling levels throughout the scanning procedures, as expected for this simple task of categorizing the easily recognized animal sounds.

#### Tonotopic map construction

As part of an earlier study (Lewis et al., 2009), a different set of participants (*n* = 7) performed a tonotopy mapping paradigm to reveal primary auditory cortices (PACs). This served to create functionally defined estimates of frequency-dependent response regions (FDRRs; or “tonotopic maps”). Regions showing tonotopic progressions (typically abutted by a mirror reversal map) were outlined for each individual and collated as a heat map, providing a probabilistic estimate of the location of PACs. Cortical volumes showing overlap of tonotopic maps by at least two individuals and that intersected with the transverse temporal gyri (Heschl's gyrus) were retained and projected onto the cortical models. The PAC foci from this prior study were largely localized to the medial two thirds of Heschl's gyri, consistent with the location of primary auditory cortices reported in anatomical studies (Rademacher et al., 2001) and functional studies (Formisano et al., 2003; Talavage et al., 2004), and thus adopted here as ROIs (Figure 2, orange) for comparative analyses.

## Results

Using an event related continuous acquisition fMRI paradigm, we imaged participants while they listened to non-conspecific animal vocalizations and action sounds, plus silent events as a baseline control. They indicated by button press as to whether the sound heard was (1) a vocalization or (2) an action sound by two alternative forced choice button response with the left hand. Both categories of sound stimuli were carefully balanced and matched overall for duration and intensity. Contrasting brain activation preferential for one vs. the other semantic category of sound source revealed a robust double-dissociation of cortical regions showing significant preference for processing animal action sounds medially in the posterior insulae [Table 2 and Figure 2, solid yellow at *p*_(uncorr)_ < 0.001, corrected to *p*_(corr)_ < 0.05 for minimum cluster size, and transparent yellow at *p*_(uncorr)_ < 0.01, *p*_(corr)_ < 0.05] vs. processing of animal (non-conspecific) vocalizations laterally in the middle and anterior superior temporal gyri (STG) [red; *p*_(uncorr)_ < 0.001, *p*_(corr)_ < 0.05]. As functional landmarks, we also charted the estimated location of primary auditory cortices (PACs) based on an overlay of tonotopically organized maps (Figure 2, orange, ROIs from our previous work; see Materials and Methods).

**Table 2:** **Group activation centroids in Talairach coordinate space for activation foci**.

**Anatomical location**	**Talairach coordinates**			**Volume (mm^3^)**
	***x***	***y***	***z***	
**VOCALIZATIONS** > **ACTION SOUNDS (*****P*** < **0.001)**				
Left aSTG	−58	−4	2	20,069
Left temporal pole	−34	10	−34	31
Right aSTG	54	−4	1	16,801
Right precentral g. (M1)	47	0	48	3303
**ACTIONS SOUNDS** > **VOCALIZATIONS (*****P*** < **0.001)**				
Left Posterior Insula	−36	−28	18	1216
Right Posterior Insula	32	−16	15	761
**ACTIONS SOUNDS** > **VOCALIZATIONS (*****P*** < **0.05)**				
Left MFG	−43	9	32	2848
Left Parietal	−42	−36	31	2011
Left pSTS	−52	−50	3	964
Left aIFS/vlPFC	−45	41	8	4908
Right aIFS/vlPFC	30	29	6	2836

*See text and Figure 2 legend for abbreviations*.

Supporting our theoretical model for semantic-level sound categorization (Figure 1), the main finding of the present study was the presence of spatially distinct cortical regions in both the left and right hemisphere auditory cortices (i.e., along or immediately surrounding the superior temporal plane) for preferentially processing the two semantically (and acoustically) distinct categories of non-conspecific biological sounds. Specifically, this included the left and right posterior insulae for animal action sounds, and the left and right aSTG for animal vocalizations. The independently derived functional estimates of PACs showed comparable levels of activation to both categories of sound (Figure 2A, orange; see histograms).

Relative to the action sounds, the vocalizations additionally showed preferential activation along the bilateral fronto-parietal operculum (Figure 2A, red) that was volumetrically contiguous with portions of the STG foci (evident in many individual datasets, and thus due at least in part to limitations in spatial resolution), plus the right lateral precentral cortex, overlapping functionally identified primary motor cortex (“M1”), which was shown to relate to left-hand button presses (see below). At lower threshold settings, brain responses to the animal action sounds additionally revealed preferential BOLD activation (Figure 2A, transparent yellow) in the left middle frontal gyrus (MFG), left parietal cortex (intraparietal sulcus), and left posterior superior temporal sulcus (pSTS), plus the bilateral anterior inferior frontal sulcus (aIFS) regions (left > right; which roughly overlapped ventro-lateral prefrontal cortices, vlPFC, more generally). This network directly overlapped with the classically defined left-lateralized mirror neuron system (Molenberghs et al., 2012). While mapping the full extent of mirror neuron systems (MNS) was beyond the scope of the present study, these results demonstrate at least partial recruitment of a left-lateralized motor-related system for processing and categorizing the animal action sounds relative to networks for processing animal vocalizations.

### Button response and right motor cortex

Participants used their left hand for button box responses with the index finger being used for vocalizations and the middle finger for action sounds. The consistent use of the left index finger for responses to the vocalization category almost certainly accounted for increased activation located along estimated right primary motor cortex (right “M1”; Figure 2A, red) in the vicinity of the left hand homunculus representation (Penfield and Boldrey, 1937). To verify this assertion, we performed three different types of analyses and control paradigms, as addressed in the Supplemental Results section (Supplemental Figure S2). In short, the three control paradigms and conditions examined demonstrated that the right precentral gyrus activation was due primarily, if not exclusively, to factors related to button response preparation and execution as opposed to acoustic signal processing related to one vs. the other category of sound.

### Sensitivity to acoustic signal attributes and perceptual features of natural sounds

In addition to category membership, a number of acoustic signal attributes distinguished the animal action sounds from vocalizations. For instance, vocalizations were characterized by greater harmonic content (e.g., Table 1), qualitatively higher rates of spectral modulation (Supplemental Figure S1C), greater spectral structure variation, and lower signal entropy measures (Table 1). Systematic exploration of these attributes, and a potential myriad of other signal attributes, of natural sounds that may lead to the observed processing double-dissociation was beyond the scope of the present study. Nonetheless, to explore *why* the two acoustic-semantic categories of natural sound may have led to differential processing, we examined various regions of interest for group-averaged parametric correlation with some of the signal attributes and perceptual features that could be parameterized along a single dimension (see Methods). We examined both single and multiple linear regression models that tested for significant correlations between BOLD signal responses within the ROIs for all sound stimuli in a given category (Figure 3). Note, that this study did not *a priori* attempt to match the range of acoustic signal attributes or perceptual ratings of the action sounds and vocalizations (e.g., clusters by category in Supplemental Figure S3). Consequently, ceiling or floor effects could in part account for some of the combinations of category-specific results. Nonetheless, significant correlations and trends persisted that served to facilitate data interpretation. In short, the left and right hemisphere auditory cortex ROIs (Figure 3A; aSTG, PAC, and posterior insulae) collectively showed a mix of parametric sensitivity to both acoustic signal attributes and perceptual features, while the left-lateralized ROIs preferential for animal action sound processing (Figure 3B; aIFS, MFG, pSTS) showed parametric sensitivity mostly to perceptual features of the sounds (Figures 3A,B highlighted cells in tables), as detailed further below.

The aSTG regions (left > right) showed a significant positive correlation of activation strength (at the temporal phase of 4–6 s after sound onset) with increasing spectral structure variation (SSV) measures of the animal vocalizations (Figure 3A), and the right aSTG and left PAC both trended toward positive linear correlation of activation strength with SSV. The vocalizations showed greater spectral modulation rates at least qualitatively (Supplemental Figure S1C), which may be correlated with SSV measures—thought detailed analyses along these lines went beyond the scope of the present study. Additionally, SSV measures were positively correlated with perceived emotional valence ratings (Supplemental Figure S3A, chart: *R*^2^ = 0.56; Steiger's Z = 3.31, two-tailed *p* < 0.01). Thus, there may be a relationship between perception of emotional cues in the animal vocalizations and aSTG activation (SSV-sensitivity), though this determination would require future study. The left PAC and bilateral aSTG regions also showed significant activation strength that demonstrated a positive co-linear correlation (Figure 3A, lower table) with the perceived understanding of intention behind the animal action sounds; the left and right aSTG also showed a positive co-linear correlation between activation to animal action sounds and the degree of perceived recognition of the animal action sounds. The posterior insulae showed a trend toward correlation between activation strength and decreasing acoustic signal entropy. Moreover, the entropy measures of the vocalizations were found to be positively correlated with the perception of understanding the intention behind the sound (Supplemental Figure S3B; chart *R*^2^ = 0.31; Steiger's *Z* = 2.20, two-tailed *p* < 0.05).

In our earlier studies using animal vocalizations, we revealed auditory cortices and STG regions that were sensitive to harmonic content (Lewis et al., 2005, 2009). In the present study, the left aSTG in this ROI-based analysis only trended toward showing a linear parametric correlation with the harmonics-to-noise ratio (HNR) values of the animal vocalizations (data not shown), but this trend in correlation did not survive with the linear regression analyses that included multiple signal and perceptual parameters (Figure 3A). This apparently weak presence of parametric sensitivity to harmonic content was likely due to both the specific location of the aSTG foci of the present study (being relatively more lateral to HNR-sensitive regions) plus the high end range of HNR values of the vocalizations (avg. +7.34 dB_HNR_), which were effectively at or near ceiling levels. The animal action sounds did not exhibit parametric HNR-sensitivity in any of the ROIs using any of the regression model configurations.

The left hemisphere aIFS, MFG, IPS, pSTS, and posterior insula ROIs (Figure 3B) were also tested for parametric sensitivity at the 4–6 and 6–8 s TR delays. Interestingly, at the later temporal phase (6–8 s after sound onset) the left aIFS, MFG, and pSTS regions all showed significant negative co-linear correlations between activation strength and degree of perceived recognition of the animal action sounds (Figure 3B, charts). The left posterior insula showed a trend for activation strength that correlated with the degree of perceived understanding of the intention behind the animal action sounds, but no correlation with perceived recognition in this or any of the other multiple linear regression analyses tested. The motor-related cortical network, however, was parametrically activated more strongly by animal action sounds that were generally deemed as more difficult to recognize. Thus, there was an apparent hierarchy of activation stages that correlated with different aspects of hearing perception, with the posterior insulae followed by left-lateralized non-auditory regions including the aIFS, MFG, and pSTS.

In sum, the above results revealed two distinct regions of auditory cortex *proper* located medial-posterior vs. lateral-anterior to primary auditory cortices (core) regions that showed preferential activation to processing animal action sounds vs. animal vocalizations, respectively. A number of left-lateralized cortical regions consistent with motor-related functions additionally showed preferential activation to the animal action sounds. An ROI analysis of parametric sensitivity to high order acoustic signal attributes and/or psychophysically assessed perceptual features revealed a gradient of response profiles for the different regions, reflective of their different functional roles in natural sound processing. In particular, these results were consistent with designating the posterior insulae as representing intermediate stages of a cortical processing hierarchy that subserves categorical processing and perception of action events at an acoustic-semantic level.

## Discussion

The present study revealed not only the existence of a major divergence of cortical regions in humans for processing action sounds vs. vocalizations, using duration and intensity matched non-conspecific natural sounds (Figure 2; and Supplemental Figure S4), but further identified a combination of both bottom-up high-order quantifiable acoustic signal attributes and top-down perceptual features that may mechanistically guide the divergence of processing along these two apparently distinct pathways. Importantly, these results support our neurobiological theoretical framework of cortical organizations mediating the perception of natural sounds at an acoustic-semantic category level (Figure 1). In particular, the results identify intermediate cortical processing stages along a hierarchical network that appears to mediate the perception of natural sounds. We selected non-human animal sounds as a sub-category of “living things,” which entailed real-world ethologically meaningful sound-producing events that most land mammals arguably need to be able to efficiently process and interpret to survive. Given our use of non-conspecific natural sounds, the cortical organizations revealed herein should apply to most, if not all, land mammalian species with hearing (and perhaps oral communication) ability.

Analogous to hierarchical and parallel processing pathways along core, belt, and parabelt auditory regions in primates (Rauschecker et al., 1995; Rauschecker, 1998; Kaas and Hackett, 2000), the separate pathway findings of the present study were consistent with representing a hierarchical processing system for extracting acoustic attributes (e.g., harmonicity, entropy, spectral structure variation, and high spectral modulation rates) and/or matching sound features to templates, which is in line with aspects of earlier models of spectral and temporal processing pathways in primates (Bendor and Wang, 2008). Presumably, acoustic information processing in primary auditory cortices (Figure 2B, orange) was subsequently routed out to the aSTG and the posterior insulae, which are regions that are not activated by lower order acoustic attributes or “simpler artificially produced sounds” such as pure tones, white noises, amplitude or frequency modulated tones and the like (Kaas and Hackett, 1998, 2000; Rauschecker and Scott, 2009). The aSTG and posterior insula regions showed parametric sensitivity to some of the perceptual features tested, including intention and emotional valence, and thus reflected processing or representation of either bottom-up acoustic signal attributes or some combination of signal attributes and top-down perceptual features. In this manner, the current findings extend “dorsal-ventral” dual-stream models of sound processing (Rauschecker and Tian, 2000; Arnott et al., 2008; Rauschecker and Scott, 2009; Hamzei et al., 2016). In particular, with regard to the ventral sound recognition stream (“what is it”), the two observed regions of the present study (bilateral posterior insulae and aSTG foci) appear to reflect portions of two major auditory pathways for purposes of sound categorization and recognition, distinguishing among different categories of biological (“living”) sound-sources at an acoustic and acoustic-semantic level—action sounds vs. vocalizations.

Notwithstanding, auditory streams directed dorsally from primary auditory cortices have often been associated with spatial-related (“where is it”) processing (*ibid*). However, the present data suggest that the posterior insulae and the more dorsally located “non-auditory” regions (aIFS, MFG, IPS) in the left hemisphere subserve functions relating specifically to sound recognition. These more dorsal “non-auditory” cortical stages showed sensitivity almost exclusively to high level perceptual features of the animal sounds (e.g., Figure 3B), such as the degree of perceived intention and/or perceived recognition. Activation of these latter stages presumably had greater dependence on factors such as personal experience and expertise, which was consistent with earlier animal studies that fail to find neurons in prefrontal regions that are sensitive to acoustic signal attributes *per se* (Cohen et al., 2007). This basic finding of a three tiered hierarchy for the processing of sounds (auditory core, posterior insulae, dorsal left-lateralized cortical network) revealed the bilateral posterior insulae as intermediate processing stages subserving categorization and recognition of real-world natural sounds at an acoustic-semantic level. Consequently, the results of the present study may reflect central processing mechanisms that were critical to the evolution and neurodevelopment of acoustic communication systems in the brain, as addressed below in the context of proposed functional roles of the posterior insulae and aSTG regions.

### Functional roles of the posterior insulae

Our earlier studies of hearing perception revealed the left and right posterior insulae as regions preferential for processing non-conspecific animal action sounds relative to other categories of sound-producing events, including human, mechanical and environmental sounds (Engel et al., 2009; Lewis et al., 2011). However, the functional role(s) of the posterior insulae in humans remained elusive. In the macaque monkey, the retroinsular (Ri) area shares at least some topological overlap with the posterior insulae of humans (Hackett et al., 2007) and thus may share some homologous functions. Area Ri has direct neuronal connections with auditory cortices as well as with second somatosensory cortex (S2) and several parietal areas (*ibid*). Moreover, preliminary electrophysiological data indicate that area Ri in the macaque has strong auditory and somatosensory responses, and robust superadditive multisensory (audio-tactile) interactions (O'Connell et al., 2004). Sound stimuli used in the present study revealed animal action sounds as having greater power in the lower frequency ranges (Supplemental Figure S1A), possibly reflective of the temporal dynamics or cadences of locomotion sounds. This finding may prove to relate to greater temporal modulation rates of action sounds more generally (Schönwiesner and Zatorre, 2009; Herdener et al., 2013), and thus is a promising topic for further study using sound stimuli that have acoustically well-controlled higher order signal attributes. Animal action sounds also had higher entropy measures than vocalizations, reflective of action sounds being significantly less “acoustically organized” on average. Together, the above neuroanatomical considerations and perceptual features suggest a possible role for the human posterior insulae in representing action sounds in relation to audio-tactile and audio-motor associations, or perhaps more generally in abstracting representations in the neural code of the temporal dynamics of observable real-world action events.

At lower threshold settings, the animal action sounds additionally evoked preferential activation involving left lateralized frontal, parietal, and pSTS regions (Figures 2A, 3B), which appeared to entail some of the classical dorsal (“where is it”) pathways (refer to Introduction). However, this more dorsally-directed system was unlikely to be recruited for purposes of sound localization relative to one's own motor spatial coordinates *per se* (e.g., to engage or avoid the sound-sources), since binaural spatial cues were removed and there was no overt spatial processing task. Nor was this dorsal system recruited for any overt linguistic-related processing (e.g., segmenting amplitude envelopes of human communication sounds) as observed in some studies of spoken language (Burton et al., 2000; LoCasto et al., 2004), since only non-verbal, non-conspecific animal sounds were used. Rather, this left-lateralized fronto-parietal plus pSTS system was more reminiscent of the location and functions ascribed to classically defined left lateralized mirror neuron systems (MNS) revealed during overt action observation (Rizzolatti and Arbib, 1998; Rizzolatti and Craighero, 2004; Molenberghs et al., 2012). MNS-like network activation in hearing perception studies in humans are thought to reflect processing that provides a probabilistic match between heard action sounds and the listener's own repertoire of sound producing actions (e.g., walking, chewing food), thereby providing a sense of meaning or intention behind the action (Buccino et al., 2001; Bidet-Caulet et al., 2005; Calvo-Merino et al., 2005; Gazzola et al., 2006; Lahav et al., 2007; Mutschler et al., 2007; Galati et al., 2008; Engel et al., 2009; Lewis et al., 2011). Ostensibly, recordings from frontal and parietal cortex neurons of the macaque monkey has revealed responsiveness when the animal either performs a goal-directed, sound-producing action or while hearing, without viewing, the same motor act (Kohler et al., 2002; Keysers et al., 2003). Thus, the primate brain appears to map complex acoustic signals and attributes of action sounds to motor- and sensory-motor-related associations as a general strategy that may subserve categorization and aspects of recognition of natural sounds.

Importantly, in the present study the non-conspecific animal action sounds, relative to vocalizations, led to relatively more robust activation of the bilateral insulae than to the left-lateralized MNS-like system. In contrast, we reported the opposite pattern when processing human action sounds relative to animal action sounds in our earlier studies of action sounds (Engel et al., 2009; Lewis et al., 2011). Thus, the posterior insulae in humans may represent stages prior to MNS-like systems for processing acoustic and perceptual attributes statistically characteristic of heard action events. While the animal action sounds were generally characterized by significantly lower harmonic content, lower spectral structure variation, and less acoustic signal organization (i.e., greater signal entropy) relative to vocalizations (Supplemental Figure S3B), they did show greater average spectral power in the 10–50 Hz range (see Supplemental Figure S1A), which is a range known to be important for conveying amplitude envelope information, such as for speech processing (Rosen, 1992). The amplitude envelope cadences representative of locomotion sounds (e.g., a horse trotting) presumably contain both low and high-order quantifiable signal attributes, such as specific envelope ranges and combinations of temporal modulation rates, that could be driving bottom-up information processing to and including pathways involving the left and right posterior insulae. This processing may in turn lead to network comparisons of acoustic temporal dynamics with the listener's own repertoire of sound-producing motoric representations in motor- and visuomotor-related networks (including left-lateralized aIFS, MFG, and IPS regions). Processing in these regions with the task of action event categorization and recognition may subsequently match, or fail to adequately match, the listeners' repertoire of sensorimotor sound producing actions to facilitate or mediate the perception of intention, and/or a sense of recognition, behind the action sound. The animal action sounds of the present study were clearly perceived as non-human, as opposed to human or self-like, and thus would arguably be less readily emulated or mimicked through motor imagery-related mechanisms. Thus, animal action sounds lead to relatively less robust recruitment of MNS-like systems for purposes of recognition, consistent with our earlier studies contrasting human and animal action sounds (Engel et al., 2009; Lewis et al., 2011). Moreover, the non-auditory dorsal network for action sounds revealed activation strength that was parametrically greater for animal actions that were generally judged by participants as being more difficult to recognize. This suggests that audio-motor association networks may have been activated to a relatively greater extent by the sounds that were harder to recognize before finally settling on a “best solution” match (Hopfield and Tank, 1985) that effectively deemed the sound as being produced by a living agent, but was not the action of a self-like (i.e., conspecific) sound-source.

Given the current interest and contentions in debates regarding MNS systems in the context of gestural origins behind the evolution of spoken language systems (Gallese and Goldman, 1998; Corballis, 1999; Arbib, 2005; Cerri et al., 2015), further study of the functional roles of the posterior insulae would be merited to address predictions made by anthropological theories. In particular, one theory hypothesizes that symbolic representations in the brain for specific natural categories, including “vocalizations” and “incidental sounds of locomotion” (in addition to “tool-use sounds”), reflect some of the earliest sound-producing categories of events that would need to have been effectively communicated orally by hominins (Hewes, 1992; Larsson, 2014, 2015). Findings of the present study, suggestive of distinct cortical processing pathways for representing action sounds as a distinct acoustic-semantic category of natural event types, may thus reflect a fundamental cortical organizational principle for mapping complex spectro-temporal attributes of natural sounds to perceptual representations, and ultimately supporting elements of acoustic communication symbolism and iconicity (Lotze et al., 2000; Monaghan et al., 2012; Perniss and Vigliocco, 2014; Lockwood and Dingemanse, 2015). Vocalizations, as another distinct acoustic-semantic category, have perhaps more obvious relationships between symbolism and oral communication, as addressed next.

### Functional roles of the aSTG

Animal vocalizations are arguably produced to convey intentional communication of some sort (e.g., distress, territorial warning, mating calls), which derive from motivational states thought to be similar across many species (Hauser, 2000; Bass et al., 2008; Lingle et al., 2012). The animal vocalizations in the present study had on average a significantly greater degree of perceived negative emotional valence in comparison to the relatively neutral valence of the animal action sounds (Table 1). The aSTG regions further showed parametric sensitivity to SSV measures, perceived intention and perceived recognition (Figure 3A, table), and also to the perceived emotional valence ratings (data not shown) which showed a correlation with SSV measures (Supplemental Figure S3A). Thus, the aSTG activation appeared to reflect both intermediate and higher stage processing of vocalizations with a functional role in representing acoustic signals containing prosodic information. Consistent with this interpretation, earlier studies have similarly reported differential activation to vocalizations conveying emotional content along the aSTG brain regions (Zatorre and Penhune, 2001; Kotz et al., 2003; Friederici and Alter, 2004; Ethofer et al., 2006; Schirmer and Kotz, 2006; Grossmann et al., 2010). Additional other brain regions commonly reported to be sensitive to emotional or prosodic cues in sound include the left anterior insula, for linking action representations to emotions (Carr et al., 2003; Wicker et al., 2003; Craig, 2009), and the left and right amygdala (Fecteau et al., 2007; Belin et al., 2008). Both of these latter regions were also preferentially activated by the vocalizations at lower threshold settings in the present study (data not shown). Therefore, the aSTG, together with a network of other brain regions, may have been extracting acoustic signal attributes that conveyed emotion and thus perhaps other forms of representation of intention behind the sound-sources. Thus, in addition to the roles that the bilateral mSTG/aSTG have in spoken language processing (Binder et al., 2000), the present results speak to a more fundamental role in processing acoustic information that conveys information regarding the sound-source's intentions or emotional state, which is another rudimentary aspect of auditory communication thought to be critical to the evolution of proto-networks for spoken language systems in hominins (Falk, 2004).

## Conclusions

In sum, the present study provides novel evidence of gross-level divergent, intermediate stage regions of auditory cortex for processing action sounds vs. vocalizations as distinct acoustic and acoustic-semantic categories of natural sounds. These pathways were consistent with neurocognitive models of embodied semantic knowledge representations and with a global theoretical framework for how ethologically valid categories of sounds may become processed as meaningful events by the brain. They were also consistent with established dual-stream (dorsal-ventral) models for auditory perception (Rauschecker and Scott, 2015); however, the “dorsal” pathways, notably including the posterior insulae of the present study, were not recruited for purposes of sound localization nor segmentation of spoken communication (language) sounds, but rather appeared to be recruited more for purposes of comparing the dynamics of spectro-temporal sound patterns to motor-related representations of actions, which may have served to help categorize and provide a sense of meaning (recognition or intentional understanding) behind the animal action sounds (Gazzola et al., 2006; Lahav et al., 2007; Pazzaglia et al., 2008). Further exploring auditory processing stages and neuronal mechanisms for representing natural sounds, including the roles of the posterior insulae relative to audio-motor association systems, will likely provide a promising approach for advancing anthropological models of language evolution. Moreover, such exploration is also likely to advance our understanding of the neurodevelopmental mechanisms underlying auditory communication, and thus impact models of acoustic signal processing in children with spoken language disorders.

## Ethics statement

All participants were consented by the laboratory principle investigator or his designated assistants, as describe in the West Virginia University approved IRB protocol (on file kc#1311130065). In short this entailed reading and signing a safety screening form for MRI safety, and a consent form, both of which are kept confidential.

## Author contributions

JL conceived and designed the study, and JL and PW interpreted the data. PW, LS, CF, HS, and JL acquired the data. All contributed to analyzing the data and revising the manuscript.

### Conflict of interest statement

The authors declare that the research was conducted in the absence of any commercial or financial relationships that could be construed as a potential conflict of interest.

## Supplementary material

Supplementary Figure S1 **Animal action sound (yellow) and vocalization (red) acoustic features and emotional valence ratings. (A)** Chart illustrating differences in the average power spectra of the two categories of sound (from Table 1). **(B)** Chart illustrating the scale rating responses (n = 15) for the perceived emotional valence for each category of sound (red curve = 29 vocalizations; yellow curve = 23 animal action sounds). Button press ratings included very negative (−2), negative (−1), neutral (0), positive (+1), and very positive (+2). **(C)** Modulation power spectra for the retained 23 animal action sounds relative to 23 (of the 29) animal vocalizations. Note that the vocalizations qualitatively reveal relatively greater power at high spectral modulation rates. Refer to text for other details.

Supplementary Figure S2 **Results from a control condition scan wherein an individual performed the main auditory paradigm, using either their (A)** right hand or **(B)** left hand for button press responses. Green cortical regions depict activation to both vocalization/index-finger and action sound/middle-finger response trial conditions relative to silence/no-response conditions (p(uncorr)<0.000001). Each trial response condition (for vocalizations and action sounds) resulted in primary motor cortex activation (green with outlines) only in the hemisphere opposite the hand used (axial slice at z = +62). Histograms show the degree of activation for the left and right hemisphere ROIs for both tasks.

Supplementary Figure S3 **Correlations between acoustic attributes and perceptual features. (A)** Chart showing the correlation between emotional ratings of animal action sounds (yellow squares) and animal vocalization sounds (red diamonds) relative to spectral structure variation measures. **(B)** Chart showing the correlation between one92s sense of perceiving the intention of the action sound or vocalization relative to the measure of entropy in the sound stimuli. Refer to Methods for further details.

Supplementary Figure S4 **Photos depicting non-human animals producing either action sounds (yellow outlines) or vocalizations (red)**. Colored brain regions (yellow and red) depict functionally distinct regions (using fMRI) that are preferential for processing the corresponding acoustic-semantic category of sound, colored respectively. Orange brain region depicts the functionally defined location of primary auditory cortex. These findings extend concepts from the ventral (“what is it”) processing pathways for sound recognition, and support a general theoretical framework for how the mammalian brain may be organized to represent natural sounds as meaningful events to the listener.
