# Supplementary figures and images for: Divergent Human Cortical Regions for Processing Distinct Acoustic-Semantic Categories of Natural Sounds: Animal Action Sounds vs. Vocalizations
Source: Front Neurosci. 2017 Jan 6;10:579. doi: 10.3389/fnins.2016.00579 (PMC5216875; doi:10.3389/fnins.2016.00579)

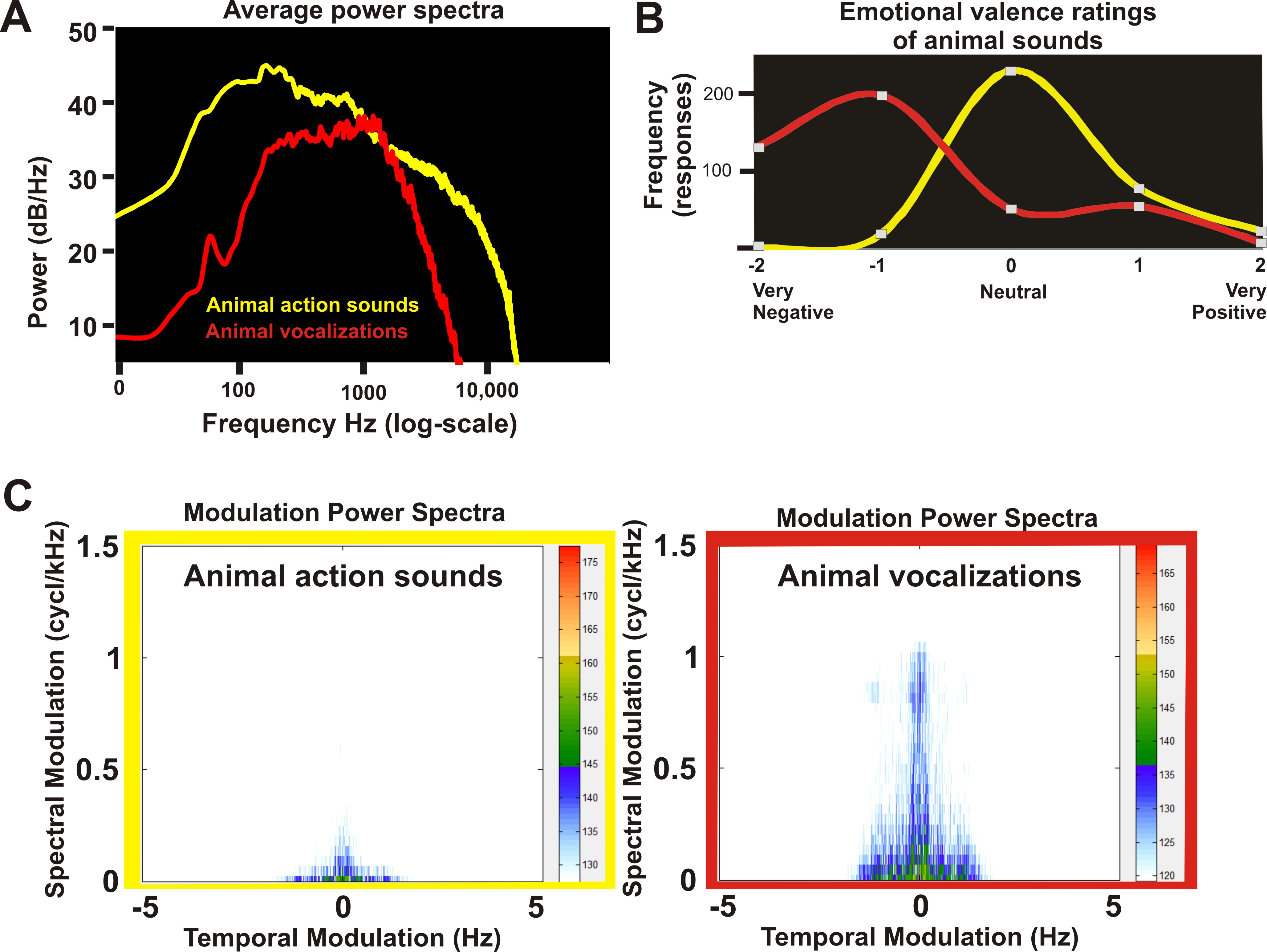

Supplement: Supplementary Figure S1 — Animal action sound (yellow) and vocalization (red) acoustic features and emotional valence ratings. (A) Chart illustrating differences in the average power spectra of the two categories of sound (from Table 1). (B) Chart illustrating the scale rating responses (n = 15) for the perceived emotional valence for each category of sound (red curve = 29 vocalizations; yellow curve = 23 animal action sounds). Button press ratings included very negative (−2), negative (−1), neutral (0), positive (+1), and very positive (+2). (C) Modulation power spectra for the retained 23 animal action sounds relative to 23 (of the 29) animal vocalizations. Note that the vocalizations qualitatively reveal relatively greater power at high spectral modulation rates. Refer to text for other details. [file Image1.jpg]

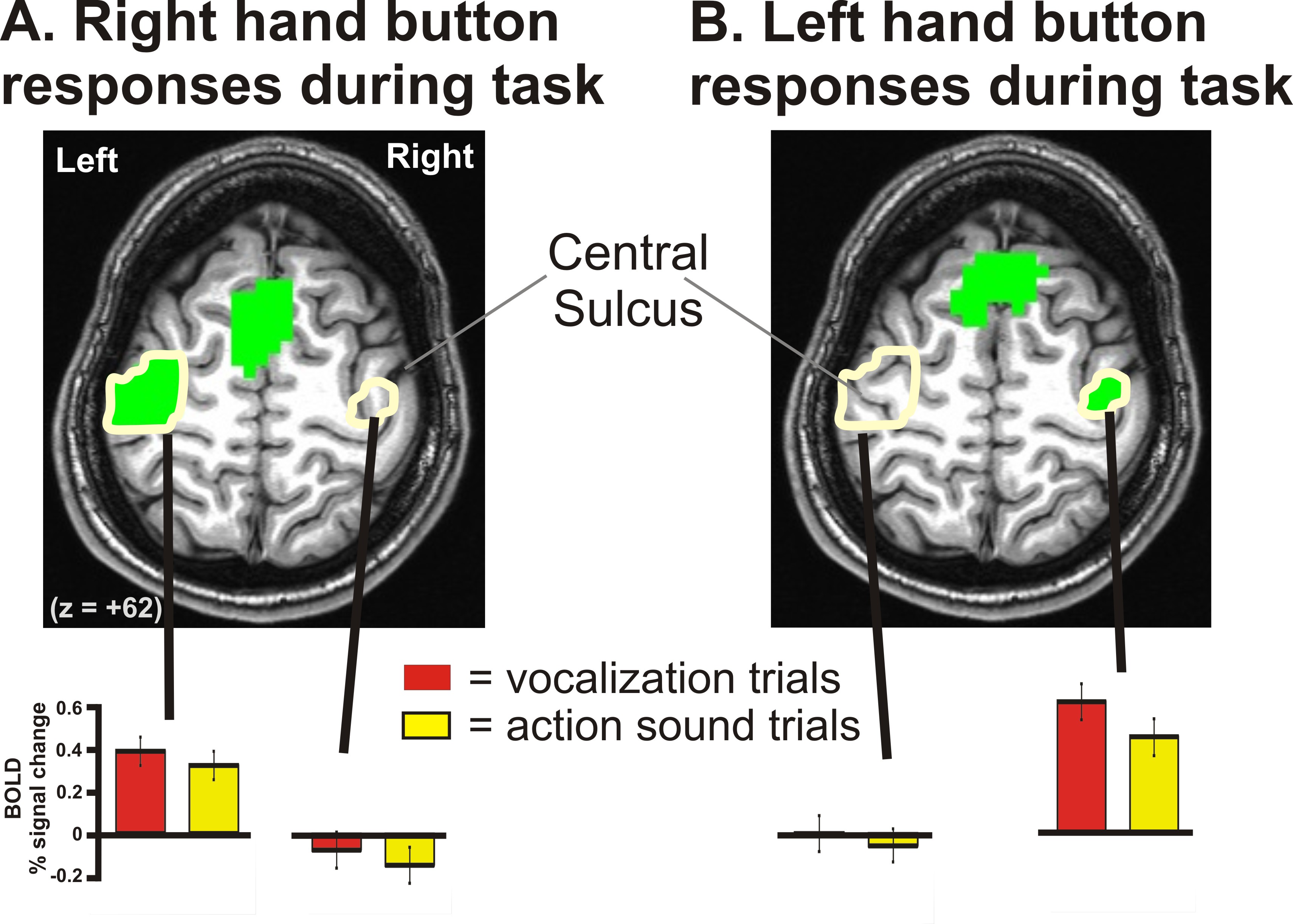

Supplement: Supplementary Figure S2 — Results from a control condition scan wherein an individual performed the main auditory paradigm, using either their (A) right hand or (B) left hand for button press responses. Green cortical regions depict activation to both vocalization/index-finger and action sound/middle-finger response trial conditions relative to silence/no-response conditions (p(uncorr)<0.000001). Each trial response condition (for vocalizations and action sounds) resulted in primary motor cortex activation (green with outlines) only in the hemisphere opposite the hand used (axial slice at z = +62). Histograms show the degree of activation for the left and right hemisphere ROIs for both tasks. [file Image2.jpg]

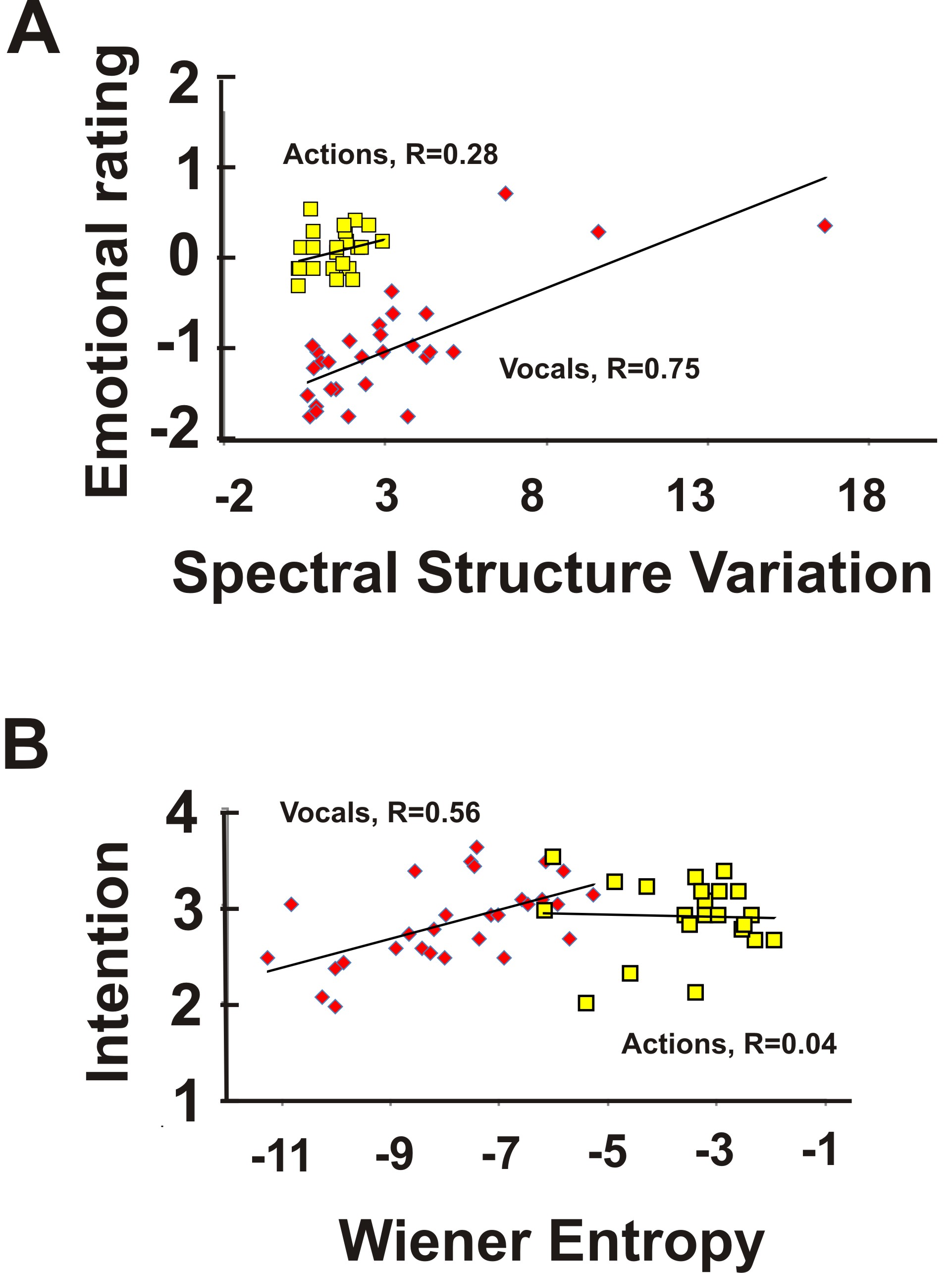

Supplement: Supplementary Figure S3 — Correlations between acoustic attributes and perceptual features. (A) Chart showing the correlation between emotional ratings of animal action sounds (yellow squares) and animal vocalization sounds (red diamonds) relative to spectral structure variation measures. (B) Chart showing the correlation between one92s sense of perceiving the intention of the action sound or vocalization relative to the measure of entropy in the sound stimuli. Refer to Methods for further details. [file Image3.jpg]

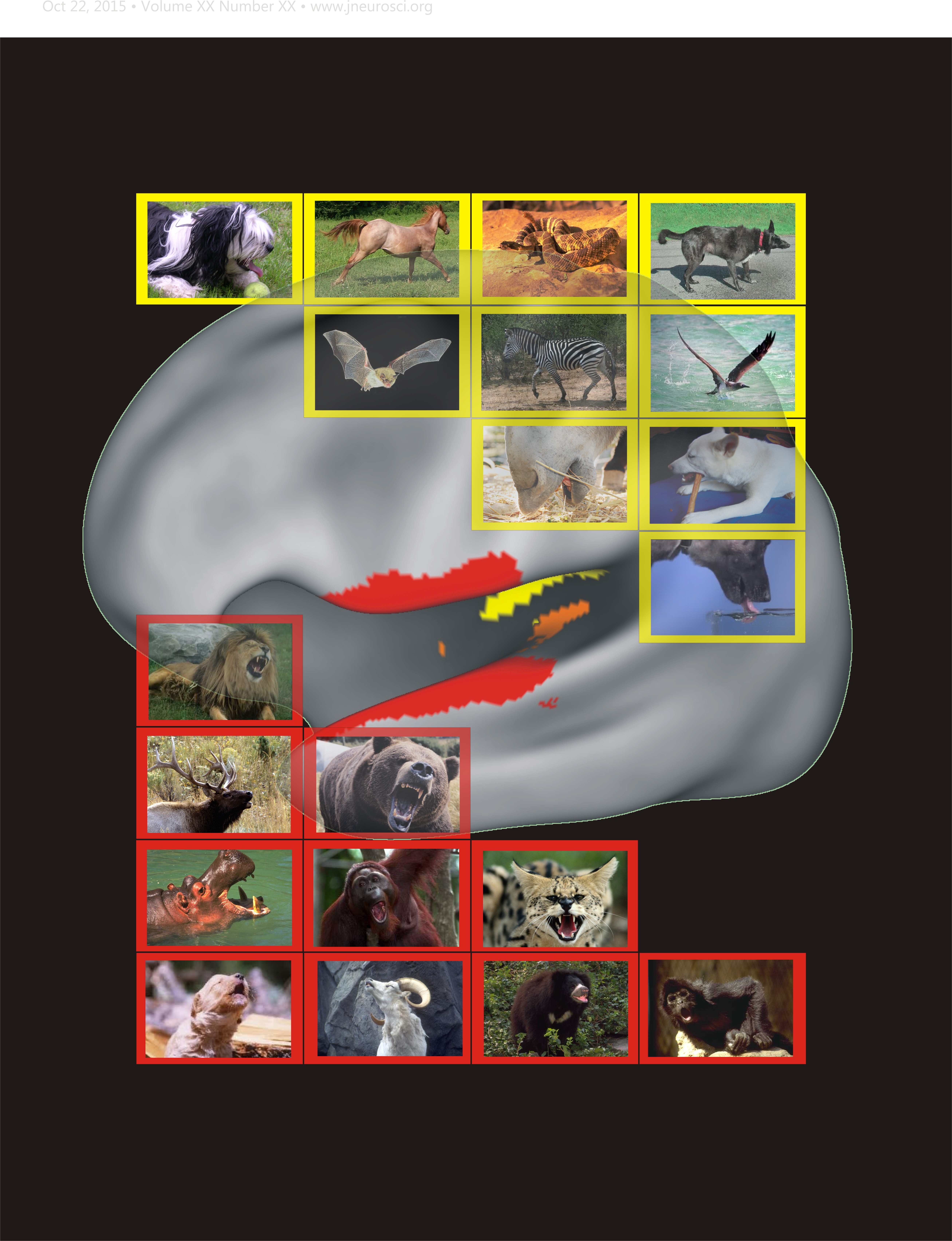

Supplement: Supplementary Figure S4 — Photos depicting non-human animals producing either action sounds (yellow outlines) or vocalizations (red). Colored brain regions (yellow and red) depict functionally distinct regions (using fMRI) that are preferential for processing the corresponding acoustic-semantic category of sound, colored respectively. Orange brain region depicts the functionally defined location of primary auditory cortex. These findings extend concepts from the ventral (“what is it”) processing pathways for sound recognition, and support a general theoretical framework for how the mammalian brain may be organized to represent natural sounds as meaningful events to the listener. [file Image4.JPEG]
