# Supplementary material for: Divergent Human Cortical Regions for Processing Distinct Acoustic-Semantic Categories of Natural Sounds: Animal Action Sounds vs. Vocalizations
Source: Front Neurosci. 2017 Jan 6;10:579. doi: 10.3389/fnins.2016.00579 (PMC5216875; doi:10.3389/fnins.2016.00579)
Supplement: Supplementary file 5 [file DataSheet1.docx]

**Supplemental Results**

The use of left index finger responses for choosing the vocalization category almost certainly accounted for activation located along the estimate primary motor cortex region (“M1”, Fig. 2A, red). To verify this assertion, we performed three different types of analyses and control paradigms. First, we examined the reaction time button responses. The average response times to correctly categorized vocalizations across all scanning runs was 2.26 ± 0.25 sec and to correctly categorized (and post hoc censored) animal action sounds was 2.26 ± 0.28 sec, showing no significant difference (ANOVA F1,157 = 0.014, p<0.90). Thus, the reaction times per se did not account for this differential activation in motor cortex. Second, two of the participants performed an fMRI paradigm wherein they were cued to generate button presses with the response box (without sound stimuli, and eyes closed) relative to resting the hand. As expected, this revealed robust motor-related activation that overlapped the right precentral cortical focus for vocalizations (data not shown). Thus, the right precentral gyrus region (“M1”) could also be activated by a motor response task in the absence of sound stimuli. Third, one participant performed the main listening task while responding with the right index finger and right middle finger for two runs and the corresponding left hand fingers for two runs. Comparison of results from these two motor task conditions demonstrated that the right precentral gyrus activation correlated exclusively with the hand used (Fig. S2, histograms). This indicated that index finger use (digit 2) led to greater intensity and expanse of primary motor cortex activation in the M1 hand representation relative to the middle finger (digit 3), consistent with earlier studies ([Lotze et al., 2000](#_ENREF_72); [Beisteiner et al., 2001](#_ENREF_11); [Indovina and Sanes, 2001](#_ENREF_55)). In this experiment, the divergent pathways for processing action sounds (posterior insulae) and vocalizations (mSTG) were qualitatively similar regardless of hand used to respond (data not shown). Together, the above control paradigms indicated that the right precentral gyrus activation was due primarily, if not exclusively, to factors related to button response preparation and execution as opposed to acoustic signal processing related to one versus another category of sound.
